# Primary Cutaneous Cryptococcosis Caused by *Cryptococcus gatti* in an Elderly Patient

**DOI:** 10.3390/tropicalmed7090206

**Published:** 2022-08-23

**Authors:** Walter Belda, Ana T. S. Casolato, Juliana B. Luppi, Luiz Felipe D. Passero, Paulo R. Criado

**Affiliations:** 1 Dermatology Department, Medical School, University of São Paulo, Clinics Hospital, São Paulo 05403-000, Brazil; 2 Laboratory of Pathology of Infectious Diseases, Medical School, University of São Paulo, São Paulo 01246-000, Brazil; 3 Institute of Biosciences, São Paulo State University (UNESP), São Vicente 11330-900, Brazil; 4 Institute for Advanced Studies of Ocean, São Paulo State University (UNESP), São Vicente 11350-011, Brazil; 5 ABC School of Medicine, Fundação Universitária do ABC (FUABC), Santo André 09060-870, Brazil

**Keywords:** cryptococcosis, cutaneous involvement, immunocompetent patient

## Abstract

According to the spread of *Cryptococcus* sp., fungal infections can be classified as primary or secondary. In primary cutaneous cryptococcosis, the fungi are restricted to the skin of the patients, without systemic involvement. The incidence of primary cutaneous cryptococcosis is high in patients with immunosuppression, and this type of infection is rarely observed in patients who are immunocompetent. In the present case report, a patient who is immunocompetent and has systemic comorbidity reported that, after skin trauma, ulcerovegetative lesions appeared in the right upper arm; the etiologic agent was identified as *Cryptococcus gatti*, serotype B. The cutaneous lesions healed completely after 5 months of fluconazole treatment.

## 1. Introduction

Cryptococcosis is an infectious disease caused by encapsulated heterobasidiomycete yeasts, namely *Cryptococcus neoformans* and *C. gatti*, which are able to affect the central nervous system, the lungs, the skin, as well as other parts of the body [1]. This fungus mainly infects individuals with immunosuppression and, thus, has been considered an opportunistic pathogen [2]. Patients with neoplasia, collagenosis, transplants, or diabetes or patients under treatment with immunosuppressant drugs are the most susceptible; contrarily, it is rare in individuals who are immunocompetent [3,4,5].

In the past, cryptococcosis was considered to be caused by different varieties of *C. neoformans*; however, based on its molecular, distribution, clinical presentation, and physiopathological characteristics, two different species have been identified as causing this fungal infection: *C. neoformans* and *C. gattii* species complexes [6]. *C. neoformans* is classified into two varieties, *C. neoformans* var. *grubii* (serotype A) and *C. neoformans* var. *neoformans* (serotype D), which can recombine, producing AD hybrids [7].

*C. neoformans* serotype A occurs worldwide and is prevalent in patients with immunosuppression, while serotypes D and AD are commonly found in European patients but have a sporadic global distribution, with their spread possibly correlated with the migration of infected birds, whose feces may contaminate the soil [8,9]. Infections caused by serotypes B and C of the *C. gattii* species are less common in patients with immunosuppression [10]; therefore, they are considered more virulent than *C. neoformans* and can cause a spread in infections even in healthy individuals [10,11,12]. Although prevalence is high in tropical and subtropical areas, human cases have also been detected in North America [11,13,14]. Both *C. neoformans* and *C. gattii* can be found in the soil, trees, bird feces, fruits, and domestic dust [6]; thus, their transmission generally occurs by inhaling yeast or desiccated basidiospores.

Human infection begins after inhalation of environmental basidiospores or desiccated yeast cells. These forms of fungi arrive in the pulmonary alveoli and in immunocompetent hosts; then, antigen-presenting cells, such as macrophages, become active and elicit cell-mediated immunity [15], ultimately eliminating the fungi or containing them in granulomas. Depending on the host–microbe relationship, cryptococci persist in a latent form within phagocytic cells and granulomas [16]. When the patient who has been infected is suppressed, dormant microorganisms reactivate and cause infection, spreading from the lungs to different tissues and systems, such as the central nervous system [17]. Cryptococcal infection can also occur through the direct inoculation of fungi forms in the skin; this form of infection is highly associated with farmer activities.

Cryptococcal infection is commonly manifested in the central nervous system (CNS) and lower respiratory tract, but the fungi can spread widely. In the CNS, cryptococcal meningitis is frequent in patients infected with HIV; symptoms are nonspecific; progress over weeks; and are similar to other causes of meningitis, including headache, fever, malaise, vomiting, memory loss, and altered mental status [18]. Furthermore, such patients may have meningismus, cranial nerve palsies, and papilledema. Depressed consciousness may be associated with an increase in intracranial pressure that can be a consequence of the obstruction of the cerebrospinal fluid by cryptococci [19]. Furthermore, patients with increased intracranial pressure may experience ocular symptoms, but yeast invasion can cause optic nerve atrophy [20]; although direct ocular infections are rare. Symptoms related to visual alterations include decreased visual acuity, sudden loss of vision, floating, and pain [21].

Pulmonary cryptococcal involvement is more frequently observed among patients with immunosuppression, and infections caused by *C. gatti* are often identified [22]. Such patients can show only an asymptomatic infection or symptomatic with the presence of pulmonary nodules, leading to acute respiratory distress syndrome [23]. Common symptoms include fever, cough, dyspnea, night sweats, malaise, and chest pain [24]. Pulmonary infection caused by *C. gattii* may be more severe than with *C. neoformans*. On radiography, nodules, mass-like lesions, cavitary lesions, inflammatory infiltrates, and pleural effusions can be observed [25], but the severity of the lesions depends on the immunological status of the patient.

Cutaneous cryptococcal infections are polymorphic, making diagnosis extremely difficult; additionally, they are classified as primary and secondary. Primary cutaneous cryptococcosis occurs when the fungi are inoculated directly in the skin through a traumatic injury, and secondary cutaneous cryptococcosis occurs when the fungi spread through the hematogenic pathway. In both clinical forms, the spectrum of lesions is wide, including ulcers, plaques, cellulitis, abscesses, pustules, papules, blisters, and nodules [26].

Other less classical organs may be affected by *Cryptococcus* sp., such as the bones. Both patients who are immunocompromised and immunocompetent can have bone involvement, especially if they are under corticosteroid treatment [27]. *Cryptococcus* can also invade and colonize the peritoneum, gastrointestinal tract, and prostate, among other organs.

In 2008, the burden of cryptococcal meningoencephalitis was estimated at around 957,900 cases worldwide per year, with more than 600,000 deaths [28,29], but its incidence decreased with the widespread availability of antiretroviral drugs. Cryptococcosis has been considered an opportunistic infection, and it is estimated that around 220,000 cases of cryptococcal meningitis occur among patients with HIV/AIDS worldwide each year, resulting in nearly 181,000 deaths; however, other patients who are immunocompromised may be susceptible to this infection, such as people with malignant neoplasms, with transplants, and under treatment with immunomodulatory drugs [30]. In Brazil, infections caused by *C. neoformans* occur in all regions, while *C. gattii* mainly infects children and young people living in the north and northeast regions of Brazil who are immunocompetent, suggesting that Brazilian States in the Northern macroregion are endemic for *C. gatti* infection [31]. However, states in the southern region, including Mato Grosso do Sul, Minas Gerais, Paraná, Rio de Janeiro, Rio Grande do Sul, and São Paulo, display only sporadic infections caused by *C. gatti* [32]; however, data about the prevalence and incidence of *C. gatti* infections in Brazil are rare [33,34].

Therapy depends on the clinical form of cryptococcosis, and on the immunological and health conditions of the patient; however, it is based on the following three main drugs: amphotericin B deoxycholate (and its lipid formulations), flucytosine, and fluconazole, administered alone or in combination. Treatment of cryptococcal meningitis is carried out in three phases: induction (2 weeks), consolidation (8 weeks), and maintenance (6–12 months) with the use of effective fungicidal drugs during the induction phase, such as amphotericin B or, alternatively, with its lipid formulation [35]. Fluconazole (400 mg/kg) is recommended during 6–12 months for patients without meningeal involvement. In patients who are immunocompetent with pulmonary involvement, flucytosine is recommended; however, voriconazole, itraconazole, or posaconazole are interesting alternatives if flucytosine is unavailable or contraindicated [36].

This case report describes a patient who presented a rare form of cryptococcosis, known as localized cutaneous cryptococcosis. Before the appearance of cutaneous lesions, he experienced traumatic events on the skin during labor in agricultural regions, which may facilitate contact between the microbe and the host, infection, and disease development. This patient did not have systemic involvement, suggesting that his immune cells restrained the yeast forms at the site of inoculation. Furthermore, such a patient was refractory to 400 mg of fluconazole administered, but the lesions healed after six months of therapy with 200 mg of fluconazole. Furthermore, this work highlighted the need for early diagnosis in patients who are immunocompetent and immunosuppressed, and show rare forms of cryptococcosis.

## 2. Case Report

**Figure 1:**
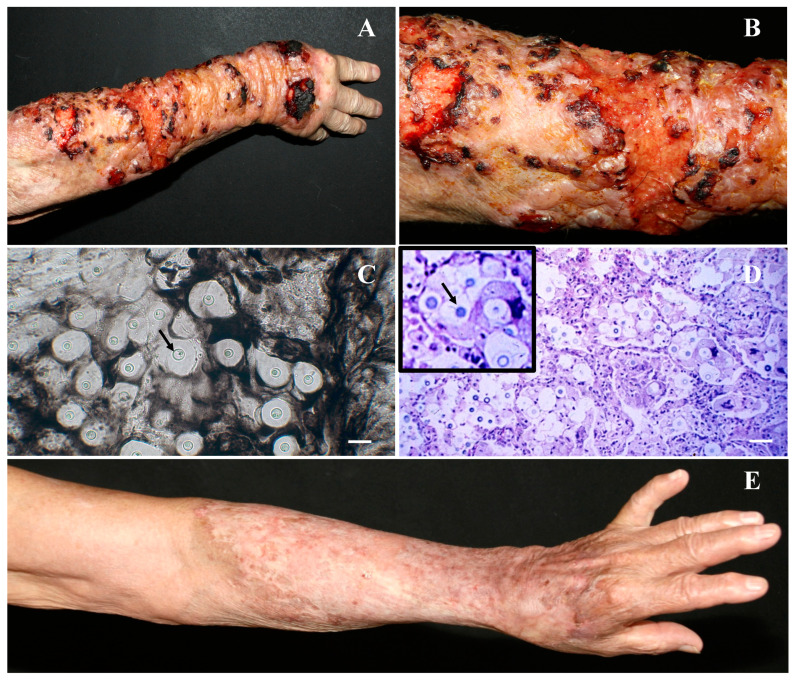
A male patient with ulcerovegetative plates, characterized by infiltrative borders, covered with hematic crusts and blisters (**A**,**B**) shown in the skin smears, stained with India Ink; capsulated fungi (**C**) and histological sections, stained with toluidine blue, showing the birefringent yeasts (**D**). The patient responded well to fluconazole (200 mg/kg), and after five months of treatment, the lesions healed completely (**E**). Bar in C and D = 10 μm.

The patient, an 80-year-old man and a retired farmer, reported the appearance of a lesion on his right forearm for six months after repeated skin injuries, which is one way the fungi could inoculate into the skin. He was admitted to the outpatient Dermatology service of the Hospital das Clínicas da Universidade de São Paulo. The lesion presented as ulcerovegetative plates, characterized by infiltrative borders, covered with hematic crusts and blisters with serous content (Figure 1A,B). According to the morphology, the lesions resembled cryptococcosis or paracoccidioidomycosis. The smears of the skin were stained with India ink, and capsulated fungi with leveduriform morphology were visualized, which were compatible with an infection caused by the genus *Cryptococcus* (Figure 1C, black arrow). A skin biopsy was collected at the margin of the lesion, and a histological study was carried out on skin sections stained with toluidine blue. A large number of birefringent intracellular and extracellular yeasts were observed, suggesting cryptococcal infection (Figure 1D, black arrow). Furthermore, skin smears were cultured in L-canavanine glycine bromothymol blue medium, allowing the isolation of the etiological agent, which was further identified by molecular biology techniques [37] as *Crytococcus gattii*, serotype B. Briefly, a 300 bp fragment from the D2 region of the 28S fungal ribosome was amplified and sequenced. Identification was performed on the NCBI GenBank website (http://www.ncbi.nlm.nih.gov/, (accessed on 16 September 2021).

From an immunological point of view, the patient was seronegative for HIV infection and for the purified protein derivative (PPD) skin test, demonstrating that he was negative for tuberculosis. All of the following parameters were normal: serum IgG, IgM, and IgA levels. No significant changes were observed in hemogram or in the number of CD4^+^ and CD8^+^ T lymphocytes, and B and NK cells. Serum protein electrophoresis and biochemical parameters were in the range of normality. The analysis of cerebrospinal fluid, collected by lumbar puncture, and radiography of the thoracic cavity revealed no significant changes in the patient, suggesting that the fungi were restricted to the skin. On the other hand, immune cells may experience age-related weakness and sometimes senescence in individuals over 60 years old [38], which in fact explain the susceptibility of this patient to *C. gattii*.

The patient was treated with fluconazole (400 mg/day), but after seven days of treatment, a significant alteration in renal function was observed, and the dose of fluconazole was reduced to 200 mg/day. Complete healing was observed after five months, and treatment was stopped (Figure 1E). The patient is under monthly monitoring, and the skin lesions have healed completely. No systemic involvement has been observed after the third month of interruption of treatment. The patient gave us authorization to publish this case report.

## 3. Discussion

Infection caused by cryptococcosis etiologic agents occurs primarily by inhalation, and fungi affect the lungs; however, in the majority of cases, infection is self-limited in patients who are immunocompetent. The hematogenic dissemination of fungi to the central nervous system has been documented and generally causes meningoencephalitis [39], but fungi can affect other organs, including the skin [40].

In disseminated cryptococcosis, skin involvement occurs in 10% of cases and can be a localized manifestation or the first signal of systemic disease. Primary cutaneous cryptococcosis is a rare condition in people who are immunocompetent, but it also occurs in patients with immunosuppression and is characterized by skin eruptions, densely parasitized with fungi; additionally, the etiologic agents do not have the ability to spread to the internal organs [41]. Primary cutaneous cryptococcosis has been detected mainly in older men who work in rural areas, are exposed to bird excrement, and have a history of local skin trauma. Furthermore, the upper extremities are the areas of the body that are most affected, and the lesions exhibit progressive centrifugal growth in exposed areas of the skin; additionally, the lesions have an ulcerative-infiltrative morphology, as observed previously [42].

However, even in patients who are immunocompetent, it is important to conduct a thorough laboratorial investigation to observe systemic involvement, considering that, in the medical literature, a case report showed that an immunocompetent patient with primary cutaneous cryptococcosis exhibited meningeal involvement, demonstrating that this patient had disseminated cryptococcosis [43]. Furthermore, another patient with primary cutaneous cryptococcosis presented a relapse in the form of fulminant meningoencephalitis after two years of being cured [39]. These reports, in fact, reinforce the importance of investigating the hematogenic dissemination of *Cryptococcus* sp., even in patients who are immunocompetent.

In fact, the patient studied here reported numerous skin traumas during his work on a farm, which may have facilitated the access and colonization of the fungi on his skin. Furthermore, all tests performed showed normal blood biochemical parameters, suggesting that internal organs were not affected by *C. gatti*; no signals of fungi dissemination were observed; and more importantly, no downregulation of host immunity was observed, suggesting that innate and acquired immune responses restricted *C. gatti* to the skin, as observed in Figure 1A,B. Although a normal number of immune cells has been found in this patient, it is important to highlight that immune functions in the skin decrease in older adults, and it may involve the activity of homing dendritic cells (DCs). Dermal DCs are able to capture antigens in the skin and to interact with lymphocytes to trigger an efficient immune response, thus being vital to the protection of the skin as well as other organs from infection [38]. In older adults, DC function is reduced in terms of migration, phagocytosis, and ability to stimulate T lymphocytes [44], which are essential to eliminating extracellular and intracellular pathogens. Although the patient in this case report showed a normal number of immune cells, the potency of his skin immunity over *C. gatti* should not be sufficient to eliminate the microbe at the time of skin injury. In addition, the T cell compartment may be in a senescent state [45] and thus not responding adequately to the aggression caused by the fungus.

Although the patient had reported skin trauma, it is important to note that inhalation is the classical mechanism of acquiring cryptococcosis, with subsequent dissemination to the central nervous system, as well as other organs, such as the skin [46]. However, the absence of fungi in other organs suggests that inoculation may be an important way to acquire primary cutaneous cryptococcosis [47]. In fact, the patient studied here reported repeated skin injuries, which can be considered a possible way to acquire a cryptococcal infection.

## 4. Conclusions

Therefore, the extensive skin lesion observed in the right superior limb of an patient who was immunocompetent could possibly have been caused by contaminated material, since he reported numerous injuries caused by sharp materials on the farm. Skin smears cultured in L-canavanine glycine bromothymol blue medium along with specific stains and molecular biology techniques led to the identification of *Cryptococcus gattii* as the etiologic agent of the skin lesion, which can infect individuals who are immunocompetent or immunosuppressed. The patient was treated with the antifungal drug fluconazole and exhibited complete remission after five months of treatment. This case report emphasizes that primary cutaneous cryptococcosis is rare in patients who are immunocompetent but may affect older adults due to the natural weakness of their immune system.

## Data Availability

Not applicable.
